# Biomarkers & Survival in Head and Neck Squamous Cell Carcinoma: A Systematic Review & Meta‐Analysis

**DOI:** 10.1002/oto2.70227

**Published:** 2026-04-14

**Authors:** Jesse Siegel, William Adams, Kent Tadokoro, Veronica Drozdowski‐Nuccio, Carol Bier‐Laning

**Affiliations:** ^1^ Department of Otolaryngology–Head and Neck Surgery Loyola University Medical Center Maywood Illinois USA; ^2^ Department of Medicine Loyola University Medical Center Maywood Illinois USA; ^3^ Southern California Permanente Medical Group Yorba Linda California USA; ^4^ Department of Otolaryngology–Head and Neck Surgery Northwestern University Feinberg School of Medicine Chicago Illinois USA

**Keywords:** Bcl‐2, biomarkers, cyclin D1, EGFR, head and neck cancer, survival

## Abstract

**Objective:**

EGFR, cyclin D1, and Bcl‐2 are proteins involved in different stages of tumorigenesis which have all been associated with poor prognosis in head and neck squamous cell carcinoma (HNSCC). In this systematic review and meta‐analysis, we aim to measure the association of each protein with survival in HNSCC.

**Data Sources:**

PubMed, Scopus, and Cochrane databases.

**Review Methods:**

A systematic review of EGFR, cyclin D1, and Bcl‐2 was conducted to determine the association between overexpression and survival in HNSCC. A weighted random‐effects meta‐analysis then measured pooled rates of overall, disease‐free, and disease‐specific survival for each protein.

**Results:**

Overexpression of EGFR was associated with worse overall mortality (HR = 1.52, *P* = .01), disease‐related mortality (HR = 1.33, *P* = .02), and disease progression (HR = 1.99, *P* < .001). Overexpression of cyclin D1 was also associated with worse overall mortality (HR = 1.93, *P* < .001), disease‐related mortality (HR = 1.57, *P* = .01), and disease‐specific mortality (HR = 1.93, *P* = .01). The association between cyclin D1 and overall mortality also remained significant in papers examining only oropharynx cancer (HR = 2.66, *P* = .03). Overexpression of Bcl‐2 was associated with worse overall mortality (HR = 1.92, *P* = .002).

**Conclusion:**

These findings support EGFR, cyclin D1, and Bcl‐2 as biomarkers which portend worse prognosis in HNSCC. Further work will be needed to understand whether measurement of these proteins can be useful in tailoring treatment strategies, and whether they can be used as targets for novel therapies.

Head and neck cancer is one of the most common categories of malignancy with over 660,000 new cases and 325,000 deaths annually worldwide, and its incidence is increasing.^1^, ^2^ Among head and neck cancers, 90% are head and neck squamous cell carcinoma (HNSCC).^2^ HNSCC is currently staged using the TNM staging system. However, clinical experience has demonstrated that tumor response to therapy and prognosis can vary widely between tumors of the same head and neck subsite with the same stage. The most well‐known example of variable response to same stage HNSCC is the group of oropharyngeal tumors associated with HPV infection which, as a group, behave in a significantly less aggressive manner compared to similarly staged HPV negative oropharyngeal tumors. The protein p16 has been identified as a biomarker for HPV‐association in oropharyngeal SCC and patients with HPV‐associated, p16 positive tumors show improved survival and better response to therapy.^3^, ^4^ This finding prompted changes to the NCCN staging guidelines and has led to the formulation of clinical trials designed to study the effectiveness of therapy de‐escalation based on p16 status.^5^ Although p16 status is the best‐known example of a tumor biomarker that informs tumor behavior, there are many research groups who have studied a variety of predictive tumor biomarkers including proteins and enzymes from a variety of regulatory pathways, genetic and epigenetic markers, as well as patient‐related factors such as immunologic markers. It is important to identify those biomarkers that are predictive of patient outcome to aid in patient understanding of prognosis and so that treatment can potentially be modified to avoid over‐ or under‐treatment. Additionally, identifying the biomarkers that show consistent findings with patient survival and other outcome measures may give clues as to the cellular pathways that are optimal targets for newly developing treatments.

Many groups are investigating a variety of biomarkers that may be predictive of outcome in patients newly diagnosed with HNSCC. Although previous reviews have evaluated the potential role of tumor biomarkers in cancer diagnosis,^6^, ^7^ a comprehensive review of biomarkers useful in predicting survival outcomes has not been performed. It is beyond the scope of one review to examine in depth all types of tumor and patient‐related markers that may be useful in predicting outcome. Therefore, the current review will summarize through a systematic approach the current knowledge of a select group of protein tumor biomarkers in hopes of identifying the most thoroughly studied biomarkers that may be useful in clinically predicting disease outcome in patients presenting with HNSCC who are treated with curative intent. The biomarkers were selected based on the number of citations that studied the biomarker, with the most commonly studied biomarkers selected for inclusion. Only protein‐level biomarkers were evaluated as the goal was to identify markers that, like p16, could be easily identified using established methods available in most clinical pathology labs. Preference was also given to biomarkers for which currently available inhibitors are available. Biomarkers were intentionally selected from differing cellular pathways to avoid redundancy. Additionally, it is hoped that identifying promising biomarkers from different pathways may give clues as to those pathways that are optimal targets for newly developing treatments.

## Methods

### Search Strategy

The study design searched PubMed, Scopus, and Cochrane databases and used the following search terms: Mouth Neoplasms; Gingival Neoplasms; Palatal Neoplasms; Tongue Neoplasms; Otorhinolaryngologic Neoplasms; Laryngeal Neoplasms; Pharyngeal Neoplasms and synonyms; Squamous Cell Carcinoma and synonyms; Biological Tumor Markers; Carrier Proteins; Tumor Suppressor Proteins; Membrane Transport Proteins; Nuclear Proteins; Disease Free Survival; Treatment Outcome.

### Inclusion Criteria

Studies were included which utilized tumor samples from patients with newly diagnosed squamous cell carcinoma of the oral cavity, pharynx, or larynx, used measurement of molecular markers at the protein level, and reported survival outcomes with hazard ratio.

### Exclusion Criteria

Studies excluded were those that used cell lines or tumor samples from recurrent tumors; studies of nasopharyngeal tumors, nonsquamous cell carcinoma of the head and neck, and skin cancers of the head and neck of any type; studies that evaluated only nonspecific DNA changes, epigenetic markers, or patient factors such as immunologic status or socioeconomic factors such as tobacco use or marital status; and those with non‐survival outcome measure or survival outcome without reporting of a hazard ratio.

### Screening

Studies (n = 1506) that reported the prognostic significance of tumor‐derived protein biomarkers and patient outcomes from 1986 to 2017 were retrieved. Two independent reviewers screened these initial search results. Abstracts and the full text were reviewed for papers meeting inclusion criteria. A total of 149 different protein biomarkers were identified. All but 11 of the biomarkers had fewer than 5 citations. Of the 11 biomarkers reported in 5 or more citations, 3 were chosen for complete statistical analysis. The decision to include the 3 selected biomarkers was to adhere to the goal of identifying the most intensively studied proteins from differing pathways for which inhibitors are available. It was beyond the scope of this study to do a complete statistical analysis of all 11 most studied biomarkers. The 3 biomarkers chosen for additional study were EGFR, cyclin D1 and Bcl‐2. The biomarker p16 was not chosen despite having the greatest number of citations since p16 has been well studied and extensively reported elsewhere.^3^, ^8^, ^9^, ^10^

### Secondary Search

A secondary, updated search was then performed of PubMed, Scopus, and Cochrane databases and used the following search terms: Genes, Bcl‐2, Cyclin D1, ErbB Receptors, and synonyms. This search was completed for the years 2017 to 2024 so as to include the most recent citations on the selected biomarkers. An additional 766 citations were retrieved. Abstracts were screened and full text reviewed for papers meeting modified inclusion criteria.

### Modified Inclusion Criteria

Studies were included that utilize tumor samples from patients with newly diagnosed squamous cell carcinoma of the oral cavity, pharynx, or larynx; include measurement of EGFR, cyclin D1, or Bcl‐2 at the protein level; and report survival outcome with hazard ratio.

### Data Extraction

**Figure 1:**
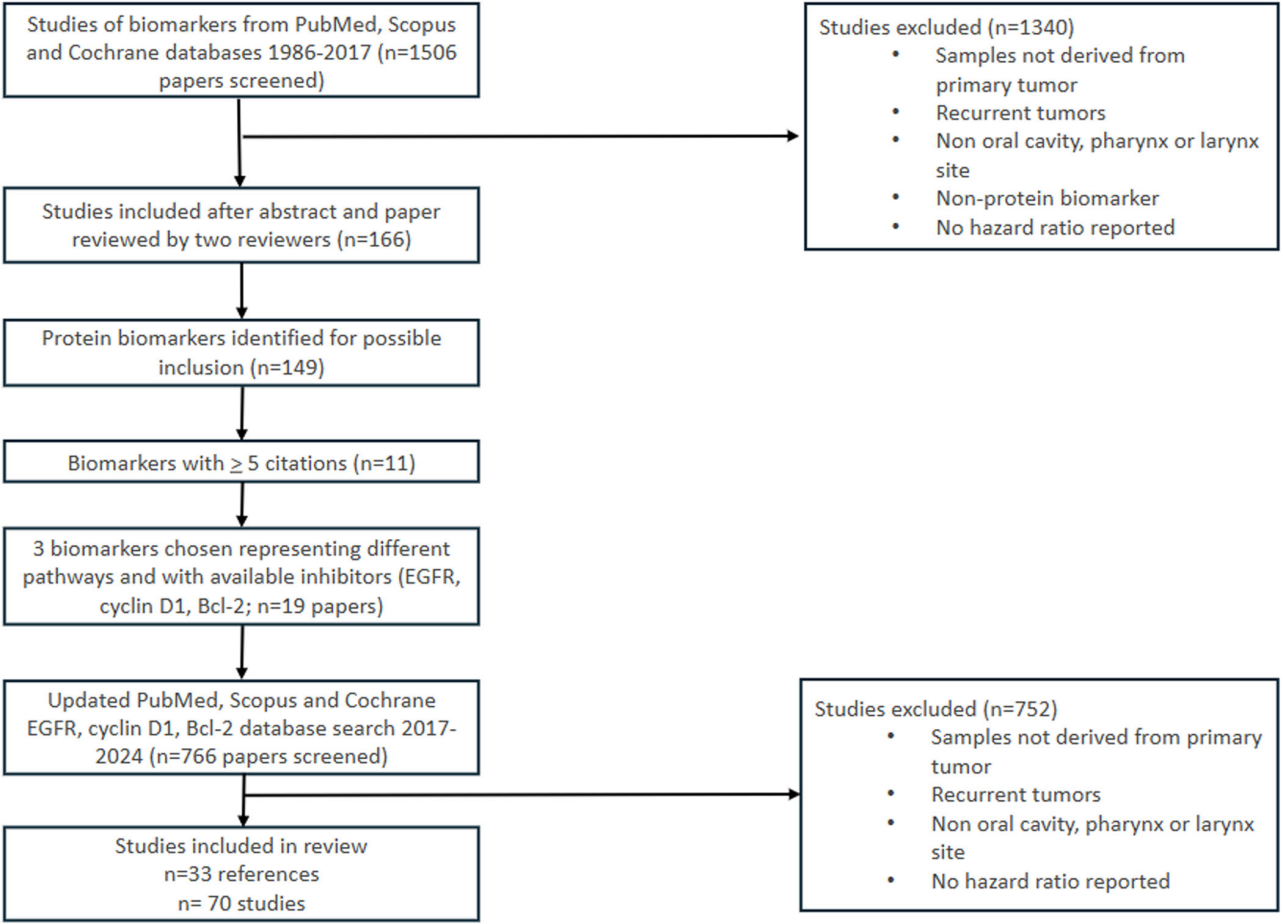
Flow diagram of study identification and review process.

The following data were included from papers meeting eligibility criteria: article citation; author list; article year; biomarker analyzed along with its sample size; hazard ratio and its standard error or 95% confidence limits; and whether the hazard ratio was reported for overall survival/overall mortality, disease free survival/disease related mortality, disease specific survival/disease specific mortality, or progression free survival/disease progression. The data also included the treatment type, tumor stage, and site. A flow diagram of the study identification and review process is included in Figure 1.

### Statistical Approach

A weighted random‐effects meta‐analysis was used to estimate the pooled rate of overall mortality, disease‐related mortality, disease‐specific mortality, and disease progression for patients with EGFR, cyclin D1, and Bcl‐2. These time‐to‐event outcomes were defined by the authors of each article used in the meta‐analysis (rather than by the current authors). Studies contributing to these estimates were weighted using their inverse sampling variance as described by Hedges and Vevea.^11^ Subsequently, results were stratified by cancer site by including the site of the cancer as a moderator in a weighted random‐effects meta‐regression model. Publication bias was evaluated visually using Light and Pillerner funnel plots.^12^ All analyses were completed using R and the metafor package.^13^, ^14^

## Results

Studies (N = 1506) that report the prognostic significance of tumor‐derived proteins and patient outcomes from 1986 through 2017 were retrieved. After limiting the updated search to EGFR, Cyclin D1, and Bcl‐2, an additional 766 citations were retrieved from 2017 to 2024. From these 2272 total citations, 33 total articles contributing 70 studies were included in the analysis (Table 1).

**Table 1:** Characteristics of Included Studies

Reference	Year	Number of patients	Biomarker	Subsite	Outcome reported
Ahmed et al^15^	2014	75	Cyclin D1	Larynx	Disease‐free survival
Ang et al^16^	2015	174	Cyclin D1	Oropharynx, hypopharynx	Overall survival
Broglie et al^17^	2015	57	EGFR	Oropharynx	Overall survival, disease‐free survival, disease‐specific survival
Bruine de Bruin et al^18^	2019	52	EGFR	Larynx	Disease‐free survival
Christensen et al^19^	2017	191	EGFR	Oral cavity	Overall survival, disease‐free survival
Christensen et al^20^	2022	93	EGFR	Oropharynx	Overall survival, disease‐free survival
Cohen et al^21^	2017	64	EGFR	Mixed	Overall survival, progression‐free survival
Csurgay et al^22^	2021	88	EGFR	Oral cavity	Overall survival, progression‐free survival
Feng et al^23^	2011	217	Cyclin D1	Mixed	Overall survival
Gupta et al^24^	2015	120	EGFR	Oral cavity	Overall survival
Gurin et al^25^	2018	77	EGFR	Oropharynx	Overall survival, progression‐free survival
Hafkamp et al^26^	2009	74	Cyclin D1	Oropharynx	Disease‐specific survival
Holgersson et al^27^	2010	39	Cyclin D1, Bcl‐2	Larynx	Overall survival
Homma et al^28^	2001	59	Bcl‐2	Larynx	Overall survival
Hong et al^29^	2010	249	EGFR	Oropharynx	Overall survival, disease‐free survival
Huang et al^30^	2012	140	EGFR	Oral cavity	Overall survival, disease‐free survival
Huang et al^31^	2012	264	Cyclin D1	Oral cavity	Overall survival, disease‐free survival
Huang et al^32^	2017	135	EGFR	Oral cavity	Overall survival, disease‐free survival
Jiang et al^33^	2018	71	EGFR	Larynx	Overall survival, disease‐free survival
Kwong et al^34^	2005	140	Cyclin D1	Oral cavity	Overall survival, disease‐free survival
Lee et al^35^	2019	37	EGFR, Bcl‐2	Larynx	Disease‐free survival
Nakano et al^36^	2016	105	EGFR	Oropharynx	Overall survival
Nichols et al^37^	2012	75	EGFR, Bcl‐2	Larynx	Disease‐free survival
Peralta et al^38^	2018	30	EGFR	Larynx	Overall survival
Perisanidis et al^39^	2012	111	Cyclin D1	Mixed	Overall survival, disease‐free survival
Plath et al^40^	2018	313	Cyclin D1	Oropharynx	Overall survival, disease‐free survival
Qian et al^41^	2015	94	EGFR	Oropharynx	Overall survival
Shah et al^42^	2009	135	EGFR, cyclin D1, Bcl‐2	Oral cavity	Overall survival, disease‐free survival
Sivarajah et al^43^	2019	278	EGFR	Oropharynx	Disease‐specific survival
Trivedi et al^44^	2011	135	EGFR, cyclin D1, Bcl‐2	Oral cavity	Overall survival, disease‐free survival
Tseng et al^45^	2021	429	EGFR	Oral cavity	Overall survival, disease‐free survival, disease‐specific survival
Yokokawa et al^46^	2020	208	EGFR	Oral cavity	Overall survival
Young et al^47^	2011	204	EGFR, cyclin D1, Bcl‐2	Mixed	Overall survival, progression‐free survival

### Overall Survival

The overall (pooled) rate of mortality was approximately 52% higher for those with EGFR (n = 20, HR = 1.52, 95% PI: 1.12‐2.07; *P* = .01). Conclusions were similar for cyclin D1 (n = 10, HR = 1.93, 95% CI: 1.41‐2.64; *P* < .001) as well as Bcl‐2 (n = 5, HR = 1.92, 95% CI: 1.26‐2.90; *P* = .002). See 2, 3, 4. For cyclin D1, cancer site was a significant moderator of these effect sizes (overall *P* = .03). That is, within the oropharynx site, those with cyclin D1 (n = 2, HR = 2.66, 95% CI: 1.10‐6.45; *P* = .03) were more likely to experience mortality. No other sites were statistically significant (all remaining *P* > .05).

**Figure 2:**
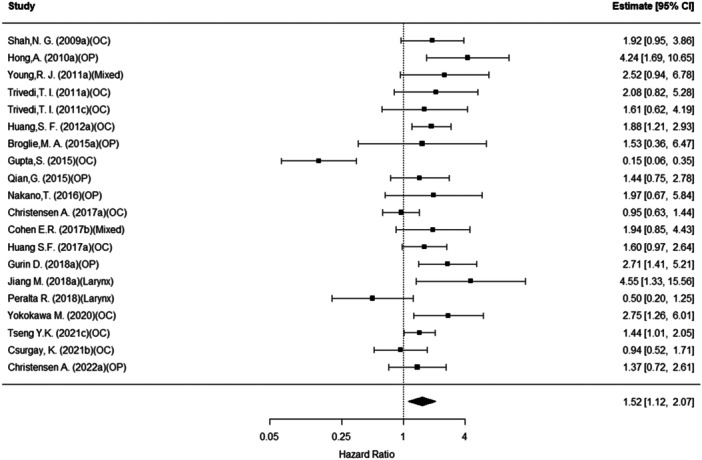
Overall mortality as a function of EGFR expression.

**Figure 3:**
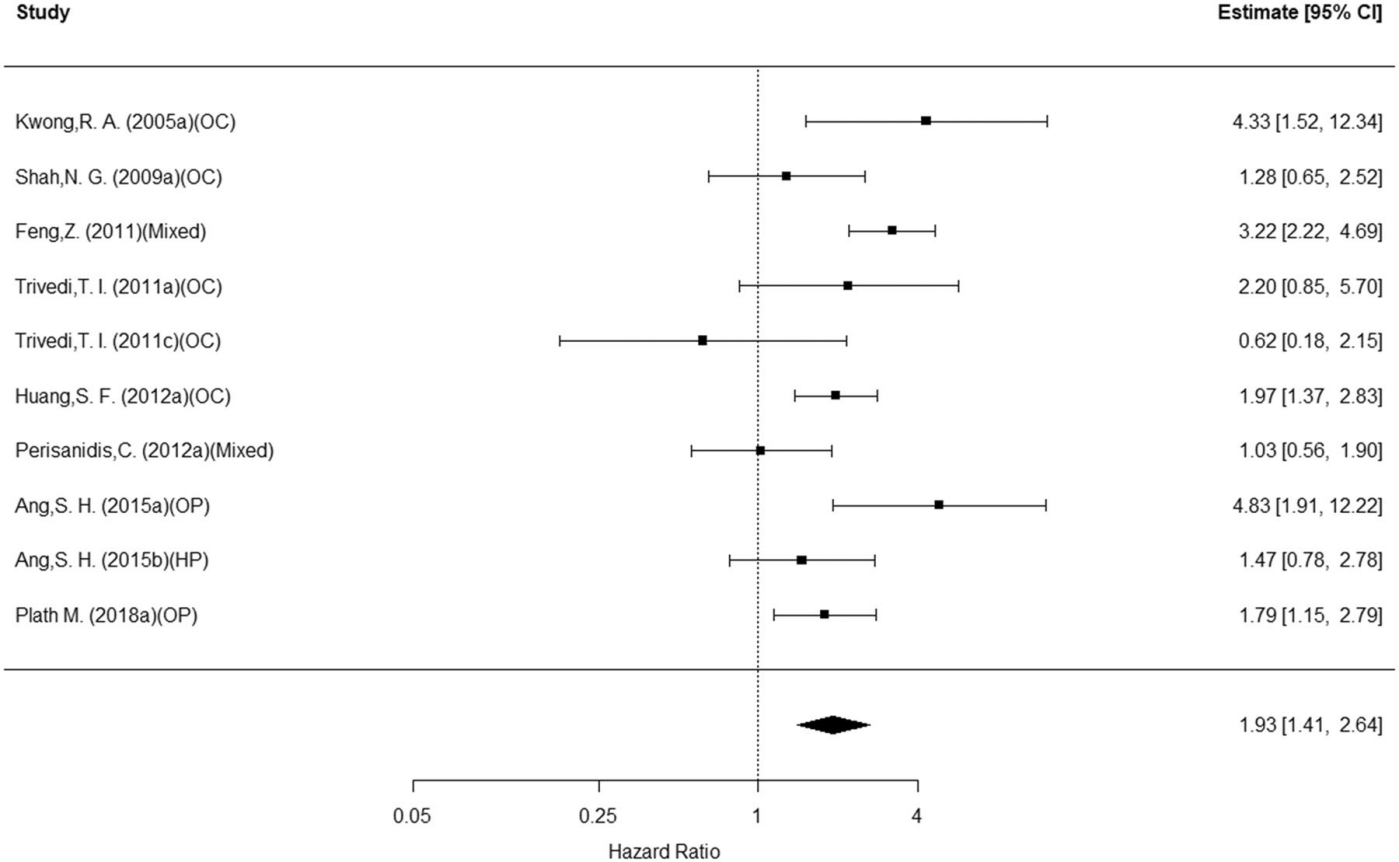
Overall mortality as a function of cyclin D1 expression.

**Figure 4:**
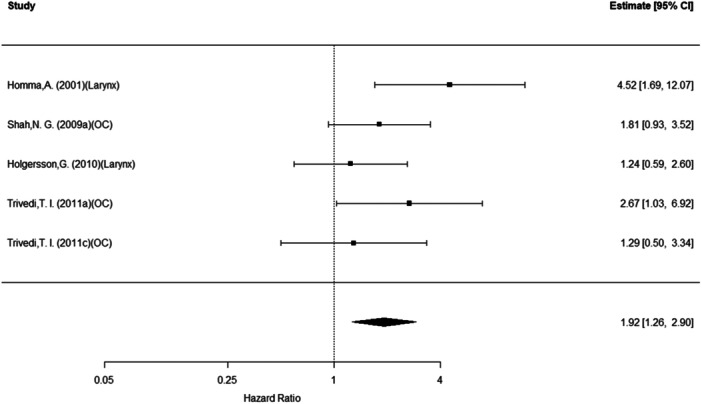
Overall mortality as a function of Bcl‐2 expression.

### Disease‐Related Mortality

The pooled rate of disease‐related mortality was also higher for those with EGFR (n = 14, HR = 1.33, 95% PI: 1.05‐1.67; *P* = .02) and cyclin D1 (n = 7, HR = 1.57, 95% PI: 1.14‐2.15; *P* = .01), while the effect for Bcl‐2 attenuated (n = 5, HR = 1.30, 95% PI: 0.90‐1.90; *P* = .17). Cancer site remained a significant moderator of these effect sizes for cyclin D1 (overall *P* = .01) and Bcl‐2 (overall *P* = .002). Within the larynx site, those with cyclin D1 (n = 1, HR = 2.44, 95% CI: 1.34‐4.45; *P* = .01) were more likely to experience the event. Conclusions were similar for the OC site where those with cyclin D1 (n = 5, HR = 1.57, 95% CI: 1.12‐2.21; *P* = .01) and Bcl‐2 (n = 3, HR = 1.80, 95% CI: 1.18‐2.74; *P* = .01) were more likely to experience disease‐related mortality.

### Disease‐Specific Mortality

Disease‐specific mortality was reported for EGFR and cyclin d1. The pooled rate of disease‐specific mortality was not statistically significant for those with EGFR (n = 3, HR = 1.66, 95% PI: 0.87‐3.16; *P *= .12) but was statistically significant for cyclin D1 (n = 2, HR = 1.93, 95% CI: 1.21‐3.09; *P* = .01). Cancer site was a significant moderator for the EGFR effect size (overall *P* = .02) where the rate of mortality remained elevated within the OP site (n = 2, HR = 2.44, 95% CI: 1.26‐4.71; *P *= .01).

### Disease Progression

Finally, disease progression was measured exclusively in 4 studies of EGFR. Here, the pooled rate of progression was approximately 99% higher for those with EGFR (HR = 1.99, 95% PI: 1.40‐2.83; *P* < .001), and cancer site remained a significant moderator of the effect size (overall *P* = .002). Among the 2 articles within the mixed site, the rate of mortality was 2.18 (95% CI: 1.22‐3.91; *P* = .01). Only 1 article was within the OC site (HR = 2.31, 95% CI: 1.13‐4.72; *P* = .02). And only 1 article was within the OP site (HR = 1.66, 95% CI: 0.94‐2.93; *P* = .08).

## Discussion

Given the prevalence, morbidity, and mortality of HNSCC, there is great interest in identifying biomarkers which are associated with prognosis and may guide options for treatment. Although other similar systematic reviews have focused on diagnosis of HNSCC, this is the first systematic review of which we are aware that examines the association between protein biomarkers and the clinically important outcome of survival. The long‐term goal of this type of work is to identify biomarkers that are measurable in a clinical lab and which in real time may assist with treatment selection and prognosis prediction for patients.

In this systematic review and meta‐analysis, we focus on 3 protein biomarkers which each contribute to different pathways of tumor survival and growth. EGFR is a surface receptor involved in cellular proliferation and differentiation, cyclin D1 is an intracellular protein involved in progression through the cell cycle, and Bcl‐2 is a mitochondrial protein involved in evasion of apoptosis. For each of these proteins, there has been evidence of an association between overexpression and poor prognosis in HNSCC, but findings have been mixed. This meta‐analysis pools results from prior work to measure associations between expression of each of these potential biomarkers with various measures of outcome. In this systematic review and meta‐analysis, we aim to assess the potential of EGFR, cyclin D1, and Bcl‐2 as prognostic biomarkers in head and neck cancer by measuring the association between overexpression of each protein with overall, disease‐related, and disease‐specific survival.

Epidermal growth factor receptor (EGFR) is a transmembrane glycoprotein involved in cell proliferation, differentiation, and survival. Activation by ligands such as epidermal growth factor or TGFα leads to conformational change which activates an intracellular tyrosine kinase and a subsequent signaling cascade for cellular proliferation and inhibition of apoptosis.^48^ EGFR overexpression is common in head and neck squamous cell carcinoma; Grandis et al found that EGFR and TGFα were overexpressed in 80‐90% of cases.^49^ The nature of the protein overexpression is more frequently a result of enhanced transcription than gene amplification.^50^ Overexpression of EGFR appears to be an early event in the process of carcinogenesis which correlates with the progression of histologic abnormalities from hyperplasia through dysplasia and carcinoma in situ, to invasive carcinoma.^51^

The prognostic value of EGFR overexpression in head and neck cancers remains somewhat controversial. Multiple studies have reported associations with EGFR overexpression and worse survival in head and neck SCCs; however, others have found discordant results and/or studied EGFR expression only in a subset of head and neck cancers.^47^, ^51^, ^52^, ^53^, ^54^ While the review by Bossi et al^53^ considered EGFR at all molecular levels, we included only studies that assessed EGFR at the protein level, as we were interested in assessing biomarkers that could be easily measured in any clinical lab, similar to the situation with p16. A meta‐analysis by Keren et al found that overall, patients with head and neck SCC with high EGFR expression had worse overall survival but commented on the large heterogeneity of findings among the pooled studies.^55^

Cyclin D1 is an intracellular protein which regulates cellular proliferation by promoting progression through phases of the cell cycle. Cyclin D1, along with cyclin‐dependent kinase (CDK) complexes, promotes cellular proliferation, cell growth, and mitochondrial activity, and inhibits DNA repair.^56^ Cyclin D1 can also function independently of CDK, interacting with a variety of transcription factors to indirectly promote cellular proliferation and growth.^56^ Amplification of the CCND1 gene and overexpression of cyclin D1 have been implicated in oncogenesis for multiple types of malignancies.^57^ The CCND1 oncogene has been shown to have a high rate of amplification in oral squamous cell carcinoma. Various gene polymorphisms have been identified that increase oncogenic potential, and chromosomal sites have been located which are sensitive to tobacco‐induced damage that can lead to amplification.^58^ However, the overexpression of the cyclin D1 protein in oral SCC is more common than can be accounted for by CCND1 amplification and mutations.^56^ Accordingly, there are multiple signaling pathways, including RTKs, PI3K, and Wnt, which increase cyclin D1 protein expression.

Increased expression of cyclin D1 has been associated with both higher T stage and locoregional lymph node metastasis in oral SCC.^59^, ^60^ There is also evidence that CCND1 copy‐number aberrations are associated with adverse pathological features including extracapsular spread and more diffuse tumor invasion pattern^61^, ^62^ Despite this large body of evidence that cyclin D1 expression is associated with more adverse features in oral SCC, evidence of its impact on prognosis has been mixed. Several studies have reported worse overall survival and disease‐free survival in oral SCC with cyclin D1 overexpression, but this has not been supported by meta‐analysis.^56^, ^59^ In addition to tumor growth and spread, cyclin D1 may decrease responsiveness to treatment. CND1 gene amplification can confer resistance to cisplatin,^63^ and 1 study showed that higher cyclin D1 expression in oral SCC was associated with worse response to treatment with chemotherapy and radiation.^64^ However, another large trial found no association between cyclin D1 levels and response to systemic therapy, and there is evidence that tumors with elevated cyclin D1 expression can be more receptive to radiotherapy, possibly due to increased proliferation rate.^65^ In addition, while cyclin D1 overexpression appears to be more common in oropharyngeal and laryngeal cancers,^56^ there is less available data on its prognosis for cancers of those sites. There is evidence of cyclin D1 overexpression being associated with worse survival in laryngeal and oropharyngeal carcinoma,^66^, ^67^ but conflicting findings have also been reported,^68^ and data are limited compared to those for oral SCC.

Bcl‐2 is a mitochondrial membrane protein which inhibits cellular apoptosis. In normal functioning, apoptosis is regulated by a balance of pro‐apoptotic proteins such as Bax and Bak, and anti‐apoptotic proteins such as Bcl‐2.^69^ Thus, overexpression of Bcl‐2 can allow for evasion of apoptosis in the setting of DNA damage, one of the hallmarks of oncogenesis which can lead to uncontrolled proliferation.^70^ Bcl‐2 overexpression can occur via either chromosomal translocations or increased transcription,^71^ and has been demonstrated as a common mechanism for tumorigenesis in head and neck cancers.^72^, ^73^ Overexpression of Bcl‐2 can also confer resistance to therapies which work by inducing apoptosis via DNA damage; several studies have reported association between upregulation of Bcl‐2 and cisplatin resistance.^74^, ^75^

Despite this, the role of Bcl‐2 overexpression as a prognostic factor is unclear. Wilson et al found that Bcl‐2 overexpression was associated with histologic dedifferentiation and worse N stage in a large cohort of patients with head and neck cancer patients.^73^ Despite this, those with Bcl‐2 overexpression had lower locoregional relapse rate and better overall survival. A 2023 meta‐analysis by Silva et al found that overexpression of Bcl‐2 is associated with worse overall survival in oral and laryngeal SCC and worse disease‐free survival in pharyngeal SCC.^76^ However, they noted that their conclusion was not reliable given the heterogeneity and high risk of bias in the original studies.

We find that EGFR overexpression was associated with overall mortality, disease‐related mortality, and disease‐specific mortality, and the only subsite that had an independent significant association with overall mortality was the oropharynx. Similarly, overexpression of cyclin D1 is significantly associated with overall mortality, disease‐related mortality, and disease‐specific mortality. Cancer site is a moderator of this association as well, and the only subsite which alone had a significant association with overall mortality was the oropharynx. Overexpression of Bcl‐2 was associated with overall mortality and disease‐related mortality, and the association with disease‐related mortality remained significant among both the oral cavity and larynx groups. In each of these cases, our findings support these proteins as effective biomarkers for poor prognosis in HNSCC.

Given the role these proteins play in carcinogenesis, there has been significant interest in targeted therapies. Cetuximab is a monoclonal antibody which binds to EGFR and competitively inhibits its activation. In 2006 Bonner et al compared cetuximab with RT to RT alone for definitive management of laryngeal SCC in a phase 3 trial and reported superior locoregional control and overall survival.^77^ However, subsequent trials have demonstrated superiority of cisplatin chemotherapy plus RT compared to cetuximab plus RT in head and neck cancers,^78^, ^79^, ^80^ and thus cisplatin+RT has remained the first‐line option for definitive systemic treatment. Trials are ongoing to elucidate cetuximab's potential use along with immunotherapy in recurrent or metastatic head and neck cancers.^80^, ^81^

Inhibition of cyclin D1‐associated pathways has been shown to inhibit tumor cell proliferation, and clinical trials are ongoing for CDK inhibitors.^56^ Inhibition of Bcl‐2 has also been a source of interest, and drugs which mimic BH3 to induce apoptosis have been developed, but toxicity remains a challenge.^71^ One BH3‐mimetic that is a selective inhibitor of Bcl‐2, venetoclax, has been approved for CLL, but its efficacy in head and neck cancers has yet to be investigated.^71^

Even if therapies targeting these proteins do not prove successful, understanding of their prognostic implications for HNSCC remains valuable. Upregulation of these proteins may themselves be drivers of oncogenesis or detectable byproducts of another source of cancer growth, as is the case for p16 in HPV‐associated HNSCC. Understanding of the importance of p16 in predicting prognosis and responsiveness to treatment has led to great interest in modifying treatment recommendations and de‐escalating therapies for p16+ oropharyngeal cancers.^82^, ^83^, ^84^ Similarly, understanding of prognostic value of other biomarkers such as EGFR, cyclin D1, and Bcl‐2 could inform attempts to escalate or de‐escalate treatment based on their presence or absence. Further work will be needed to determine whether testing for these biomarkers can be used as mitigating or aggravating factors when determining treatment strategies for HNSCC.

In this meta‐analysis we are limited by heterogeneity of measurement of the proteins of interest. We only include papers which use immunohistochemistry to detect these proteins, but variation in methods and in cutoffs for high or low expression could be a confounding factor when pooling results of multiple studies. More accurate prognostication may be possible with qualitative measurements of protein biomarkers.^53^ Additionally, we are limited by availability of studies which stratify by cancer sites. Many papers in our systematic review included multiple sites, so we were limited in our ability to differentiate between sites in measuring prognostic value of each biomarker. The treatment regimens and stage of disease also differed between papers and was not always limited to 1 treatment type or stage within each paper. These factors may confound the results. Additionally, the specific definition of outcomes may differ slightly between papers, so this may also add to heterogeneity.

The pooled estimates in this study may be biased towards hazardousness due to publication bias. That is, Light and Pillerner funnel plots^12^ revealed there were few null studies included in the meta‐analysis. This may be because the study primarily included nonrandomized, retrospective studies (ie, where authors are more likely to suppress null findings), or it may be related to the lethality of head and neck squamous cell carcinoma (ie, meaning there will be few null studies of EGFR, cyclin D1, and Bcl‐2 in the literature). Future research studies may wish to include more null conclusions in their meta‐analyses.

Despite these potential shortcomings, we believe that our findings support EGFR, cyclin D1, and Bcl‐2 as important targets for future study. Further measurement of their associations with survival in HNSCC, particularly if stratified across different anatomical sites, may provide a basis for counseling patients on prognosis, tailoring treatment strategies using current modalities, and potentially guiding development of new targeted therapies.

## Author Contributions

**Jesse Siegel**, acquisition and interpretation of data, drafting and critical revisions of the manuscript and approval of final manuscript; **William Adams**, study design, statistical analysis and interpretation, drafting and critical revisions of the manuscript and approval of final manuscript; **Kent Tadokoro**, acquisition and interpretation of data, approval of final manuscript; **Veronica Drozdowski‐Nuccio**, acquisition and interpretation of data, approval of final manuscript; **Carol Bier‐Laning**, study design, acquisition and interpretation of data, drafting and critical revisions of the manuscript, approval of final manuscript.

## Disclosures

### Competing interests

None.

### Funding source

None.
